# Investigation of antibiotic susceptibilities of *Brucella* Strains isolated from various clinical samples in eastern Turkey

**DOI:** 10.1186/s40001-021-00527-5

**Published:** 2021-06-16

**Authors:** Esra Gültekin, Muhammet Hamidullah Uyanık, Ayşe Albayrak, Selçuk Kılıç

**Affiliations:** 1 Department of Medical Microbiology, Faculty of Medicine, Erzincan Binali Yıldırım University, Erzincan, 24100 Turkey; 2 Department of Medical Microbiology, Atatürk University Faculty of Medicine, Erzurum, 25240 Turkey; 3 Department of Infectious Diseases and Clinical Microbiology, Atatürk University Faculty of Medicine, Erzurum, 25240 Turkey; 4 Department of Microbiology Reference Laboratories and Biological Products, General Directorate of Public Health, Ankara, 06100 Turkey

**Keywords:** Antimicrobial susceptibilities, Biotyping, *Brucella*, *E* test

## Abstract

**Background:**

Brucellosis is a worldwide zoonotic disease that causes serious public health problems. This study aimed to identify *Brucella* strains isolated from various clinical samples by conventional and molecular methods and to determine antimicrobial susceptibilities against doxycycline (DOX), streptomycin (STR), ciprofloxacin (CIP) and rifampicin (RIF) by the gradient strip (*E* test) test method.

**Methods:**

A total of 87 *Brucella* strains isolated from various clinical specimens between 2004 and 2018 were included in this study. While four of the 87 strains included in the study were identified only at the genus level, the remaining 83 strains were identified at the species level by the Real-Time Multiplex PCR **(**M-RT-PCR) method and conventional methods were used for biotyping.

**Results:**

According to molecular identification results, 83 strains were identified as *B. melitensis* by the M-RT-PCR method, with 82 strains identified as *Brucella melitensis* biovar (bv) 3 and one as *B. melitensis* bv 1 according to the conventional biotyping method. Among the antibiotics studied, CIP was found to be the most active agent according to the minimum inhibitory concentrations (MIC)_90_ values. This was followed by DOX and STR, respectively. While all of the isolates were sensitive to CIP, DOX and STR, 18 (20.7%) strains were found to be moderately susceptible to RIF, with the highest values of MIC_50_ and MIC_90_.

**Conclusions:**

In our study, all strains were identified as *B. melitensis*. DOX, STR, CIP and RIF used in the treatment of brucellosis were found to be effective.

## Background

The *Brucella* species is a zoonotic infectious agent that can be transmitted to humans by direct contact with body secretions by impaired skin, inhalation and conjunctiva, as well as the consumption of meat, milk and milk products of animals, such as infected sheep, goats, cattle and pigs. Although human-to-human transmission is rare, it can also be transmitted through sex, blood transfusion and breast milk [1]. The incubation period of brucellosis, which is a systemic infectious disease, is 2–3 weeks. It begins with general signs of infection, progresses with septicemia and can be seen in different clinical forms effecting many organs [1, 2].

With the appropriate clinical manifestations, the diagnosis of the disease occurs by serological tests and the isolation of the agent [1, 3]. Although definitive diagnosis is isolation of bacteria from culture in brucellosis, serological methods are mostly preferred, due to the difficulties in isolating the agent most of the time, especially in chronic cases, the risk of laboratory infection, and the delayed results [4]. Although many serological methods can be used in the diagnosis of brucellosis, Rose Bengal Test (RBT) and Standard Tube Agglutination (STA) tests are the most widely preferred methods all over the world. Enzyme-Linked Immunosorbent Assay (ELISA) is a reliable serological method that can quickly detect specific immunoglobulins (IgG, IgM, IgA) used in the diagnosis of brucellosis with high sensitivity and specificity [5]. The isolation, identification and biotyping of the *Brucella* species are very important for both epidemiological studies and eradication programs. Information about the species and biotype distribution of *Brucella* is important as it will contribute to the follow-up of biotypes and vaccine strains in Turkey as well as the selection of optimal strains used in serological diagnosis [6].

The World Health Organization (WHO) recommends a combination of doxycycline (DOX) and rifampicin (RIF) for at least 6 weeks for the treatment of brucellosis. Alternatively, it recommends a combination of streptomycin (STR) for 2–3 weeks and DOX for 6 weeks [7]. Tetracyclines cannot be used in children younger than 8 years. For this reason, 6 weeks of trimethoprim/sulfamethoxazole (TMPSMZ) and 3 weeks of STR or 7–10 days of gentamicin may be used in children under the age of eight, and treatment protocols such as RIF and TMP/SMZ or RIF and an aminoglycoside for 6 weeks are recommended. Tetracyclines cannot be used in pregnancy, either. Fetal toxicity has been reported in pregnant women treated with STR. For this reason, TMP/SMZ during pregnancy and alternatively, at least 45 days of RIF are recommended. It is recommended to use DOX for 8 weeks or longer in the treatment of spondylitis, which is one of the complications of brucellosis, and adding RIF or TMP/SMZ, which crosses the blood–brain barrier well, to DOX-STR regimens for at least 6–8 weeks or longer in the treatment of neurobrucellosis. In the treatment of endocarditis, it is recommended to add RIF or co-trimoxazole to a combination of DOX and aminoglycoside for at least 8 weeks [1].

Although *Brucella* isolates are generally thought to be sensitive to antibiotics, there have been cases reported with antibiotic resistance and relapse. Resistance to drugs used in the treatment of brucellosis is a particularly important problem in low socioeconomic areas of developing countries, where tuberculosis is endemic. This problem raises concerns about developing drug resistance to long-term tuberculosis medications in brucellosis treatments [2]. Therefore, it is important to follow the sensitivity of antibiotics widely used in the treatments. This study aimed to identify the *Brucella* strains isolated from various clinical samples by conventional and molecular methods and to determine antibiotic susceptibilities against DOX, STR, ciprofloxacin (CIP) and RIF by the gradient strip (*E* test) method.

## Methods

A total of 87 *Brucella* strains isolated from various clinical specimens at the Atatürk University Medical Faculty, Medical Microbiology Laboratory between 2004 and 2018 were included in this study. The samples included in the study were isolated from patients from Erzurum and 10 surrounding provinces in the east of Turkey. *Brucella* strains were isolated from blood (*n* = 84), bone marrow (*n* = 2) and cerebrospinal fluid (CSF) (*n* = 1).

The strains were identified at the species level using colony morphology, Gram staining, growth characteristics, oxidase, catalase, motility testing and polyvalent antisera agglutination methods. Strains previously isolated and identified as *Brucella* spp. were stored in 10% skimmed milk and maintained at − 80 °C until conventional biotype, molecular typing and antibiotic susceptibility testing were performed. Strains were subcultured twice before beginning the study.

### Biovar determination by conventional methods

For the identification of *Brucella* spp., conventional biotyping methods were used, including the CO_2_ requirement, H_2_S production, urease activity, sensitivity to thionin and basic fuchsin dyes (20 and 40 μg/ml), lysis by Tbilisi phage and agglutination with monospecific A and M antisera [8].

### Molecular typing of *Brucella* species

The real-time multiplex PCR (M-RT-PCR) method was used for molecular identification at the genus and species levels. Identification of *Brucella* spp., *B. melitensis* and *B. abortus* was performed with this method, simultaneously. All of the strains included in the study were inoculated into Serum Dextrose Agar (SDA) medium, and the plates were incubated at 35 °C for 48 h in a 5% CO_2_ atmosphere. To obtain bacterial DNA, the QIAamp® DNA Mini and Blood kit was used. Molecular identification of *Brucella* from the obtained bacteria, DNA IS711 element BMEI1162 (GenBank, NC003317) was used for *B. melitensis*; IS711 element (GenBank, AF148682) specific alkB gene was used for *B. abortus*; bcsp31 gene (GenBank, NM20404) region was used for *Brucella* spp., and also TaqMan probs (TaqMan, Palo Alto, USA) were used. M-RT-PCR LightCycler® FastStart DNA Master HybProbe (Roche Diagnostics, Germany), 1.5 mM MgCl_2_ (Roche Diagnostics, Germany), forward primer (3 nM) (Sigma-Genosys, USA), reverse primer (100 nM), each 16 μl multiplex PCR reaction mixtures containing 100 nM from a probe were prepared and 4 μl templates were added. Then, amplification was performed on the LightCycler 480 PCR (Roche Diagnostics, Germany). Amplification was performed on a totally 45 cycles with the following steps; after 10 min of denaturation at 95 °C followed by 15 s at 95 °C and 1 min at 60 °C. The results were obtained using the LightCycler 96 assay program, which evaluates the presence or absence of logarithmic fluorescence signal increase at the wavelength appropriate for each probe. In each study, at least one positive and DNA-free negative control was used.

### Determination of antimicrobial susceptibility

Minimum inhibitory concentrations (MIC) of DOX, STR, RIF and CIP were determined by the *E* test method (Biomerieux®, France). The *E* test strips were stored at − 20 °C until the time of use. An inoculum was prepared in 0.5 McFarland turbidity in Muller–Hinton broth (Oxoid®) from each *Brucella* strain and applied with a sterile swab to Muller–Hinton agar plates and supplemented with 5% sheep blood. The *E* test strips were placed on the plate and incubated at 37 °C for 48 h [2]. The determination of MIC was evaluated according to the recommended reference ranges for *Brucella* species in the Guidelines for Clinical and Laboratory Standards (CLSI) for DOX and STR, and for the reference ranges recommended for slow-growing bacteria (*Haemophilus*) for RIF and CIP [9]. In each step of our study, *B. melitensis* biovar (bv) 1 (16 M), *B. melitensis* bv 3 (Ether), *B. abortus* bv 1 (NCTC10093), *B. abortus* bv 3 (Tulya), *B. suis* bv 1 (1330), *E. coli* ATCC 25922 and *S. aureus* ATCC 29213 were used for quality control. The lowest antibiotic concentration that inhibits the growth of bacteria was accepted as MIC. In addition, inhibition of 50% of the isolates as MIC_50_ and inhibition of the 90% of the isolates as MIC_90_ were accepted.

## Results

The study included 87 *Brucella* strains isolated from different patients (30 female and 57 male). Samples had been sent from different clinical departments (Table 1).

**Table 1:** Distribution of samples from clinical departments

Clinical departments	Samples
Paediatrics	33
Infectious Diseases	23
Internal Medicine	23
Neurology	2
Anaesthesia and Reanimation	1
Emergency	1
Obstetrics and Gynaecology	1
Orthopaedics and Traumatology	1
Physical Medicine and Rehabilitation	1
General Surgery	1
Total	87

According to the results of the M-RT-PCR method, 83 strains were identified as *B. melitensis*. The remaining 4 strains were identified only at the genus level and were detected as *Brucella* spp. According to the results of conventional biotyping, 82 of 83 strains were identified as *B. melitensis* bv 3 and one as *B. melitensis* bv 1. According to the results of the antibiotic susceptibility test performed by the *E* test method, all strains were susceptible to DOX, STR and CIP. Of the 87 isolates, 69 (79.3%) were sensitive to RIF and 18 (20.7%) were intermediate sensitive (MIC > 1 μg/ml). The antibiotic susceptibilities of *Brucella* strains are shown in Table 2.

**Table 2:** Antibiotic susceptibility distribution of *Brucella* strains (μg/ml)

Antibiotics	Sensitivity	Intermediate	Resistant	The limit of sensitivity
DOX	87	–	–	≤ 1
STR	87	–	–	≤ 8
RIF	69	18	–	≤ 1*
CIP	87	–	–	≤ 1

*RIF Intermediate resistance: 2, Resistance: ≥ 4

According to MIC_90_ values, CIP was the most effective agent against *Brucella* strains, followed by DOX and STR. The highest MIC_50_ and MIC_90_ values were determined in RIF. The MIC ranges and MIC_50_ and MIC_90_ values of the various antimicrobial agents used against isolates in this study are given in Table 3.

**Table 3:** MIC ranges and MIC_50_ and MIC_90_ values of the various antimicrobial agents against *Brucella* species

Antibiotics	Number of strains	MIC range μg/ml	MIC_50_ μg/ml	MIC_90_ μg/ml
DOX	87	0.016–1	0.047	0.19
STR	87	0.064–0.75	0.19	0.5
CIP	87	0.002–0.25	0.064	0.125
RIF	87	0.008–2	1	1.5

## Discussion

The most common etiological agent in human brucellosis is *B. melitensis*, followed by *B. abortus*, *B. suis* and *B. canis*, respectively [10].

In different studies *B. melitensis* is the most commonly isolated strains in *Brucella* genus. These data is supported by the researchers in various countries. Baykam et al. [11], Parlak et al. [12], Hashim et al. [3] and Kılıç et al. [13] found the rate of *B. melitensis* to be the most commonly isolated species at 88.1%, 97.3%, 97.6% and 99.4% in their studies, respectively. At the same time, in some studies, *B. melitensis* was the only species isolated [14–16]. All of the strains isolated in our study were also *B. melitensis*.

In Turkey the most frequently isolated species is *B. melitensis*, and *B. abortus* is rarely seen. Among the most frequent *B. melitensis* strain, *B. melitensis* bv 3 was isolated, while bv 2 was not reported at all [4]. In the study conducted by Bodur et al. [17], 95.12% of the strains isolated were *B. melitensis* bv 3 and 4.88% were *B. melitensis* bv 1. In the study conducted by Denk et al. [10], 93.75% of the isolated strains were identified as *B. melitensis* bv 3 and 6.25% as *B. melitensis* bv 1.

In another study conducted by Çerekci, all 187 strains suspected as *Brucella* species were identified as *B. melitensis* bv 3 [4]. Similarly, the most commonly isolated bv in our study was *B. melitensis* bv 3. Out of the 83 strains examined by conventional biotyping methods, 82 (98.79%) of them were identified as *B. melitensis* bv 3 and one strain was identified as *B. melitensis* bv 1.

The main problems encountered in brucellosis are treatment failure and relapse. Relapses usually occur in the first year after infection and, in most cases, the reasons are inadequate dosage, short-term treatment and inability of the patient to comply with treatment. In addition, the pharmacokinetics and immune status of the cells may play an important role in the development of relapses. The failure of treatment is often related to the pharmacokinetic and pharmacodynamics of antibiotics rather than resistance. Since the *Brucella* can survive in phagocytic cells, long-term combined therapy, as well as selective agents, must be able to penetrate both macrophages and remain active in the acidic environment [18]. Antimicrobials commonly used in the treatment of brucellosis are DOX, RIF, STR, (trimethoprim sulfamethoxazole) TMP/SMZ and quinolones [19, 20].

Antimicrobial susceptibility tests for *Brucella* strains, except for life-threatening conditions such as treatment failure, relapse, brucella meningitis and endocarditis, are generally not recommended in routine microbiology laboratory research as they require level 3 biosafety measures and are a high risk for laboratory workers [21]. In addition, there is no standardized antimicrobial susceptibility test for *Brucella* species established by the CLSI [2]. However, in 2006, the National Committee for Clinical Laboratory Standards (NCCLS) published a standardized method for determining the MIC values of potential bioterrorism agents. In this method, it was reported that TET/DOX, TMP/SMZ and STR can be studied by the microbroth dilution method for *Brucella.* [22]. However, in various studies, MIC values for RIF and CIP were determined based on the reference ranges recommended for slow growing bacteria (*Haemophilus*) in the CLSI guideline [2, 9, 14, 21, 23]. However, EUCAST’s breakpoint tables for interpretation of MICs and zone diameters do not contain any information regarding the breakpoint values related with the *Brucella* species [24]. In our study, the susceptibilities of *Brucella* species to various antimicrobials were evaluated by CLSI criteria [9].

Apart from the microbroth dilution method, agar dilution and *E* test methods are also used to determine MIC values. The disc diffusion method is not recommended for antimicrobial susceptibility testing [25]. In our study, the *E* test method, which is reliable, repeatable, practical and also requires less labour and time than other tests, was used [2, 21].

TET are the most effective and preferred agents for the treatment of brucellosis and are recommended for most treatment combinations, except in some special cases [10]. DOX is the most widely used TET derivative due to its ability to be administered once or twice a day, superior pharmacokinetic properties and less gastrointestinal side effects than TET [1, 16]. In various studies in different countries, MIC_90_ values of DOX against *Brucella* species were found to be 0.25 μg/ml [3, 14, 23]. On the other hand, this value was considered quite high for MIC_90_: 32 in a study in China [26]. Some studies in Turkey found DOX to be the most active agent against *Brucella* strains according to the MIC_90_ value [10, 16, 17]. Unlike these studies, Etiz et al. reported that DOX was not as effective as TMP/SXT according to MIC values [2]. In our study, DOX was the most active agent after CIP against *Brucella* strains according to the MIC_90_ value.

STR is known as one of the most active agents in the treatment of brucellosis. Although side effects, such as ototoxicity and nephrotoxicity, limit its wide and parenteral use, it is preferred for the good results in bone-joint involvement [16]. In work carried out by Altun et al. [27], considerably higher MIC values of STR (0.125 to 256 μg/ml) and 8.3% of strains was resistant to STR. However, in many studies, MIC ranges (0.064–8 μg/ml) are still within the sensitivity range [2, 3, 14, 16, 18, 20, 23, 28–30]. In our study, the MIC range was 0.064–0.75 μg/ml and was consistent with the previous studies. According to the results of these studies, it can be seen that the antimicrobial agent can be used safely in terms of failures caused by resistance problems.

CIP is an important alternative in the treatment of brucellosis because of its excellent oral bioavailability and that it reaches high concentrations in phagocytic cells [23]. In the study conducted by Yamazhan et al. [19], although high MIC values were determined for CIP, low MIC values were reported in many other studies [2, 11, 23, 28, 29, 31, 32]. Kılıç et al. [18] reported that CIP showed good activity when compared to TET and Ayaşlıoğlu et al. [30] reported that it was as effective as TET. In their study, Bodur et al. [17] determined that CIP was the most active agent after DOX according to the value of MIC_90_. In our study, CIP was found to be the most active agent according to the MIC_90_ value. However, CIP is not recommended in monotherapy due to the lack of bactericidal activity at intracellular acidic pH, high recurrence rates and the risk of developing resistance to all fluoroquinolones in the community [23, 30, 32].

RIF is a potent antibiotic for the treatment of brucellosis. It has been widely accepted as the first choice in treating brucellosis. Several studies have reported that RIF has excellent activity against *Brucella* species, a good intracellular penetration and a significant synergism with combination treatments [16, 21]. Intermediate resistance rate to RIF changed between 2.1 and 75% of *Brucella* strains in Turkey [2, 12, 18, 20, 21, 30, 31]. In our study, 20.7% of the *Brucella* strains had intermediate resistance to RIF. However, in Turkey Etiz et al. [2] and Kaya et al. [20] found resistance to RIF by *Brucella* strains to be 2.00% and 2.94%, respectively.

In the study conducted by Abdel-maxoud [14] in Egypt, possible resistance to RIF was detected in 19% of the strains, while the study by Deshmukh [23] in Qatar found a higher resistance rate of 48%. There is potential for *Brucella* strains to resist RIF. The use of RIF as a long-term antitubercular agent in the treatment of tuberculosis simultaneously with brucellosis in many regions of the world may lead to the emergence of moderately susceptible and resistant *Brucella* strains. At the same time, the extensive use of RIF in the treatment of brucellosis may cause resistance to *M. tuberculosis* [2, 16].

## Data Availability

Data sharing is not applicable to this article as no data sets were generated or analysed during the current study.

## Abbreviations

RBT: Rose Bengal Test
STA: Standard Tube Agglutination
ELISA: Enzyme-Linked Immunosorbent Assay
DOX: Doxycycline
RIF: Rifampicin
STR: Streptomycin
TET: Tetracycline
TMP/SMZ: Trimethoprim sulfamethoxazole
CIP: Ciprofloxacin
*E* test: Gradient strip
M-RT-PCR: Real-time multiplex PCR
SDA: Serum Dextrose Agar
MIC: Minimum inhibitory concentrations
CLSI: Clinical and Laboratory Standards

## References

[1] Corbel MJ (2006). Brucellosis in humans and animals.

[2] Etiz P, Kibar F, Ekenoglu Y, Yaman A (2015). Characterization of antibiotic susceptibility of *Brucella* spp. isolates with E-test method. ACM.

[3] Hashim R, Ahmad N, Mohamed Zahidi J, Tay BY, Mohd Noor A, Zainal S (2014). Identification and in vitro antimicrobial susceptibility of *Brucella* species isolated from human brucellosis. Int J Microbiol.

[4] Çerekci A, Kılıç S, Bayraktar M, Uyanık MH, Yaşar E, Esen B (2011). Comparison of conventional methods and real-time multiplex polymerase chain reaction for identification and typing of *Brucella* isolates of human origin. Mikrobiyol Bul.

[5] Gültekı̇n E, Uyanık MH, Albayrak A, Aksoy O, Ayyıldız A (2012). Bruselloz tanısında kullanılan çeşitli serolojik yöntemlerin karşılaştırılması. Türk Mikrobiyol Cem Derg.

[6] Şimşek H, Erdenliğ S, Oral B, Tülek N (2004). Typing-biotyping of *Brucella* isolates of human origin and their epidemiologic evaluation. Klimik Derg.

[7] Joint FAO/WHO expert committee on brucellosis. World Health Organ Tech Rep Ser. 1986;740:1–132. 3097967

[8] Alton GG, Jones LM, Pietz DE (1975). Laboratory techniques in brucellosis.

[9] Clinical and Laboratory Standards Institute (2010). Performance standards for antimicrobial susceptibility testing. 20: M 100-S20.

[10] Denk A, Demırdag K, Kalkan A, Ozden M, Cetınkaya B, Kılıc SS (2015). In vitro activity of *Brucella melitensis* isolates to various antimicrobials in Turkey. Infect Dis.

[11] Baykam N, Esener H, Ergönül Ö, Eren Ş, Celikbaş AK, Dokuzoğuz B (2004). In vitro antimicrobial susceptibility of *Brucella* species. Int J Antimicrob Agents.

[12] Parlak M, Güdücüoğlu H, Bayram Y, Çıkman A, Aypak C, Kılıç S (2013). Identification and determination of antibiotic susceptibilities of *Brucella* strains isolated from patients in Van, Turkey by conventional and molecular methods. Int J Med Sci.

[13] Kılıç S, Ivanov IN, Durmaz R, Bayraktar MR, Ayaşlıoğlu E, Uyanık MH (2011). Multiple-locus variable-number tandem-repeat analysis genotyping of human *Brucella* isolates from Turkey. JCM.

[14] Abdel-Maksoud M, House B, Wasfy M, Abdel-Rahman B, Pimentel G, Roushdy G (2012). In vitro antibiotic susceptibility testing of *Brucella* isolates from Egypt between 1999 and 2007 and evidence of probable rifampin resistance. Ann Clin Microbiol Antimicrob.

[15] Marianelli C, Graziani C, Santangelo C, Xibilia MT, Imbriani A, Amato R (2007). Molecular epidemiological and antibiotic susceptibility characterization of *Brucella* isolates from humans in Sicily, Italy. J Clin Microbiol.

[16] Bayram Y, Korkoca H, Aypak C, Parlak M, Çıkman A, Kilic S (2011). Antimicrobial susceptibilities of *Brucella* isolates from various clinical specimens. Int J Med Sci.

[17] Bodur H, Balaban N, Aksaray S, Yetener V, Akıncı E, Çolpan A (2003). Biotypes and antimicrobial susceptibilities of *Brucella* isolates. Scand J Infect Dis.

[18] Kılıç S, Dizbay M, Hizel K, Arman D (2008). In vitro synergistic activity of antibiotic combinations against *Brucella melitensis* using E-test methodology. Braz J Microbiol.

[19] Yamazhan T, Aydemir Ş, Tünger A, Serter D, Gökengin D (2005). In vitro activities of various antimicrobials against *Brucella melitensis* strains in the aegean region in Turkey. Med Princ Pract.

[20] Kaya O, Akçam FZ, Yaylı G (2012). Investigation of the in vitro activities of various antibiotics against *Brucella melitensis* strains. Turk J Med Sci.

[21] Sayan M, Kılıc S, Uyanık MH (2012). Epidemiological survey of rifampicin resistance in clinic isolates of *Brucella melitensis* obtained from all regions of Turkey. J Infect Chemother.

[22] Clinical and Laboratory Standards Institute (2006). Performance standards for antimicrobial susceptibility testing. 26: M 100-S16.

[23] Deshmukh A, Hagen F, Sharabasi OA, Abraham M, Wilson G, Doiphode S (2015). In vitro antimicrobial susceptibility testing of human *Brucella melitensis* isolates from Qatar between 2014–2015. BMC Microbiol.

[24] The European Committee on Antimicrobial Susceptibility Testing. Breakpoint tables for interpretation of MICs and zone diameters. Version 11.0; 2021. http://www.eucast.org.

[25] Shapiro DS, Wong JD, Murray BE, Pfaller MA, Tenover FC, Yolken RH (1999). *Brucella*. Manual of clinical microbiology.

[26] Xu XL, Chen X, Yang PH, Liu JY, Hao XK (2013). In vitro drug resistance of clinical isolated *Brucella* against antimicrobial agents. Asian Pac J Trop Med.

[27] Altun B, Hasçelik G, Gür D (2009). In vitro activity of tigecycline against *Brucella* spp.. Med J Trakya Univ.

[28] Turkmani A, Ioannidis A, Christidou A, Psaroulaki A, Loukaides F, Tselentis Y (2006). In vitro susceptibilities of *Brucella melitensis* isolates to eleven antibiotics. Ann Clin Microbiol Antimicrob.

[29] Ozhak-Baysan B, Ongut G, Ogunc D, Gunseren F, Sepin-Ozen N, Ozturk F (2010). Evaluation of in vitro activities of tigecycline and various antibiotics against *Brucella* spp.. Pol J Microbiol.

[30] Ayaşlıoğlu E, Kılıç S, Aydın K, Kılıç D, Kaygusuz S, Ağalar C (2008). Antimicrobial susceptibility of *Brucella melitensis* isolates from blood samples. Turk J Med Sci.

[31] Turan H, Arslan H, Uncu H, Azap Ö, Şerefhanoğlu K (2007). In vitro activity of tigecycline against *Brucella* strains: a comparative study with doxycycline, ciprofloxacin and rifampin. Turk J Infect.

[32] Eşel D, Sümerkan B, Ayangil D, Telli M (2004). *Brucella melitensis* suşlarının antibiyotik duyarliliklarinin belirlenmesinde agar dilüsyon ve E-test yöntemlerinin karşilaştirilmasi. ANKEM Derg.

